# The single-point insulin sensitivity estimator (SPISE) index is a strong predictor of abnormal glucose metabolism in overweight/obese children: a long-term follow-up study

**DOI:** 10.1007/s40618-021-01612-6

**Published:** 2021-06-17

**Authors:** I. Barchetta, S. Dule, L. Bertoccini, F. A. Cimini, F. Sentinelli, D. Bailetti, G. Marini, A. Barbonetti, S. Loche, E. Cossu, M. G. Cavallo, M. G. Baroni

**Affiliations:** 1 Department of Experimental Medicine, Sapienza University of Rome, Rome, Italy; 2 Department of Medical Sciences and Public Health, University of Cagliari, Cagliari, Italy; 3 Pediatric Endocrine Unit, Ospedale Pediatrico Microcitemico “A. Cao”, Cagliari, Italy; 4 Department of Clinical Medicine, Public Health, Life and Environmental Sciences (MeSVA), University of L’Aquila, L’Aquila, Italy; 5 Neuroendocrinology and Metabolic Diseases, IRCCS Neuromed, Pozzilli, IS Italy

**Keywords:** Childhood obesity, Insulin resistance, Impaired glucose regulation, Insulin-sensitivity index, SPISE, Screening

## Abstract

**Purpose:**

To investigate the relationship between the single-point insulin sensitivity estimator (SPISE) index, an insulin sensitivity indicator validated in adolescents and adults, and metabolic profile in overweight/obese children, and to evaluate whether basal SPISE is predictive of impaired glucose regulation (IGR) development later in life.

**Methods:**

The SPISE index (= 600 × HDL^0.185^/Triglycerides^0.2^ × BMI^1.338^) was calculated in 909 overweight/obese children undergoing metabolic evaluations at University of Cagliari, Italy, and in 99 normal-weight, age-, sex-comparable children, selected as a reference group, together with other insulin-derived indicators of insulin sensitivity/resistance. 200 overweight/obese children were followed-up for 6.5 [3.5–10] years, data were used for longitudinal retrospective investigations.

**Results:**

At baseline, 96/909 (11%) overweight/obese children had IGR; in this subgroup, SPISE was significantly lower than in normo-glycaemic youths (6.3 ± 1.7 vs. 7 ± 1.6, *p* < 0.001). The SPISE index correlated positively with the insulin sensitivity index (ISI) and the disposition index (DI), negatively with age, blood pressure, HOMA-IR, basal and 120 min blood glucose and insulin (all *p* values < 0.001). A correlation between SPISE, HOMA-IR and ISI was also reported in normal-weight children. At the 6.5-year follow-up, lower basal SPISE—but not ISI or HOMA-IR—was an independent predictor of IGR development (OR = 3.89(1.65–9.13), *p* = 0.002; AUROC: 0.82(0.72–0.92), *p* < 0.001).

**Conclusion:**

In children, low SPISE index is significantly associated with metabolic abnormalities and predicts the development of IGR in life.

## Introduction

Overweight and obesity in childhood are conditions epidemically spread worldwide, and the dramatic increase of their incidence in the last decades has become a relevant public health issue around the world [1]. Data show that 17.9% of European children were overweight or obese during the period 2006–2016. The prevalence estimate of obesity was 5.3%, with highest values reported in the Southern European countries [2]. The increasing prevalence in children comes with escalations in both current childhood and future adulthood morbidity and mortality [3], but also in concomitant costs [4].

Early onset obesity is an independent risk factor for the development of insulin resistance and type 2 diabetes (T2D)[5], and insulin resistance represents the most common metabolic disorder associated with obesity [6, 7]. Insulin resistance and T2D are well known independent risk factors for cardiovascular diseases [8].

The pathogenesis of insulin resistance in children is a multi-factorial process and obesity is the most prevalent risk factor [9]. Also genetic predisposition [10], gestational diabetes [11], children born small for gestational age (SGA)[12], rapid post-natal weight gain [13], premature birth [14] and smoking during pregnancy [15], increase the risk of insulin resistance during childhood.

Not all obese children are insulin resistant, and insulin resistance can also occur in non-obese children [16]. The prevalence of insulin resistance in obese children varies from 33.2 to 52.1% [17–19], depending on the method and cut-off used to define insulin resistance.

Indeed, the identification of accurate tools for risk stratification in obese/overweight children is a crucial step for designing strategies to prevent metabolic diseases and their complications later in life. However, standards for assessing insulin resistance in children in the clinical setting are still lacking, and insulin-derived indexes of insulin resistance/sensitivity are affected by methodological issues, such as the poor standardization of insulin measurement [16, 20–22]. Among the non-insulin-derived indices, the single-point insulin sensitivity estimator (SPISE) index is a lipids and BMI-based index of insulin sensitivity that showed better accuracy than other indicators, such as its forerunner TG/HDL-C ratio and HOMA-IR, in the prediction of metabolic syndrome and has been recently validated in adolescents and adults [23, 24]. To date, the usefulness of the SPISE index has not been investigated in a pediatric population. Furthermore, no prospective data exists on the predictive value of this parameter on insulin resistance and glucose abnormalities.

Therefore, aims of this study were (i) to investigate the relationship between the SPISE index, glyco-metabolic profile and insulin sensitivity in a large population of children, with and without obesity, and (ii) to evaluate whether basal SPISE is predictive of the development of impaired glucose metabolism later in life.

## Research design and methods

### Study population

For the purposes of this study, we carried out a cross-sectional and a longitudinal retrospective investigation.

For the cross-sectional phase of this study, we analysed the SPISE index from data obtained in 909 overweight or obese children (median age [interquartile range]: 10 [8–13] years) consecutively recruited at the Paediatric Endocrine outpatient clinics of the Paediatric Hospital for Microcitaemia, Cagliari, Italy. Study participants were selected among all those referring to the clinic for the presence of excess bodyweight, as for indication of the general practitioner; main exclusion criteria were the presence of endocrine disorders or genetic syndromes, including syndromic obesity. After visit, all children with overweight or obesity were instructed to follow an educational program including dietary and lifestyle modifications.

To provide a reference range in children with normal metabolic status, the SPISE index was also calculated in 99 normal-weight healthy children (median age [interquartile range]: 11 [9–12.9] years) with age and sex distribution comparable to the first cohort. These normal-weight children, without any endocrine, cardiovascular, gastrointestinal or renal disorder, were recruited in the same clinical setting and selected among those referring to the Paediatric outpatients’ clinic for undergoing routine clinical assessment. The enrolment of the whole study population occurred between May 2007 and May 2010.

200 out of 909 overweight/obese children were followed-up between 2013 and 2016, with a median (range) follow-up duration of 6.5 (3.5–10) years, and data collected were used for the longitudinal investigation (see reference [25] for description of this cohort).

### Clinical and biochemical evaluations

At the baseline, all the study participants (*n* = 1008) underwent medical history collection, clinical examination and fasting blood sampling. Children with fasting blood glucose less than 126 mg/dl underwent oral glucose tolerance test (OGTT) at both the baseline and follow-up visit, following clinical recommendations for children (1.75 g of glucose administered per kg bodyweight, up to 75 g) [26]. Blood glucose and insulin concentrations were measured at the baseline and after 120 min from the oral glucose load. The presence of an abnormal glucose metabolism (AGM), in terms of impaired glucose regulation (IGR: impaired fasting glucose, IFG, impaired glucose tolerance, IGT) or diabetes mellitus, was diagnosed according to criteria from the ADA Standards of Medical Care in Diabetes 2021[27].

Systolic and diastolic blood pressure (SBP, DBP, mmHg) was measured after 10-min rest and the average value of three measurements was recorded for the analysis.

Overweight, obesity and standard deviation score body mass index (SDS–BMI) were defined according to the Italian growth charts for height, weight and BMI in people aged 2–20 years, more than one standard deviation (SD) of BMI defined overweight and more than 2 SD defined obesity [28].

Study participants were classified as pre-pubertal or pubertal according to the Tanner stage for pubertal development (pre-puberal, for Tanner’s stage I: boys with pubic hair and gonadal stage I, girls with pubic hair stage and breast stage I; puberal for Tanner’s stages ≥ II–V: boys with pubic hair and gonadal stage ≥ II and girls with pubic hair stage and breast stage ≥ II) [29]

### Laboratory procedures

Blood samples were obtained from the antero-cubital vein after 12-h fasting for evaluating routine biochemistry and metabolic profile, including blood glucose (FBG, mg/dL), insulin (FSI, IU/mL), aspartate aminotransferase (AST, IU/L), alanine aminotransferase (ALT, IU/L), total cholesterol (mg/dL), high-density lipoprotein cholesterol (HDL-C, mg/dL), triglycerides (mg/dL) and uric acids (mg/dL).

Plasma glucose levels were measured by glucose oxidase method (Autoanalyzer, Beckman Coulter, USA) and insulin concentration by radio-immunoassay (DLS-1600 Insulin Radioimmunoassay Kit, Diagnostic System Laboratories Inc., Webster, Texas, USA) on samples separated, frozen and stored at −80 °C until the analyses.

AST, ALT, total cholesterol HDL-C, triglycerides and uric acids were measured in the local laboratory by standard methods. Low-density lipoprotein cholesterol (LDL-C) value was obtained using the Friedewald formula.

Insulin sensitivity and resistance were estimated by calculating validated indirect indexes. Insulin sensitivity was assessed by the SPISE index (= 600 × HDL^0.185^/Triglycerides^0.2^ × BMI^1.338^) with fasting HDL-C and Triglycerides concentrations expressed in mg/dL and BMI as kg/m^2^ [23] and the Insulin Sensitivity Index (ISI: 10 000/√(fasting glucose × fasting insulin × mean glucose (OGTT) × mean insulin (OGTT)); the Homeostasis Model Assessment for Insulin Resistance (HOMA-IR: fasting insulin (microU/L) × fasting glucose (mg/dl)/405) and secretion (HOMA-β%: (360 × fasting insulin (microU/L))/(fasting glucose (mg/dl) − 63) and the insulin secretion adjusted for insulin resistance (Disposition Index, DI: ratio of the change in insulin to the change in glucose from 0 to 30 min (ΔI0–30/ΔG0–30) × 1/fasting insulin) [30–33]were also calculated.

### Ethics standards

The study protocol was reviewed and approved by the local Ethics Committee and conducted in conformance with the Helsinki Declaration. Informed written consent was obtained from the children or their legal guardians before all the study procedures.

### Statistics

All the analyses were performed using the SPSS statistical package, version 25.0. Values are shown as mean ± standard deviation (SD), median (interquartile range, IQR) or percentage, as appropriate. Skewed variables were log-transformed before the analyses. Differences between two independent groups were compared by Student's *T* test for continuous variables and by *χ*^2^ test for categorical parameters. Correlations were estimated by Pearson’s and Spearman’s tests, in relation to the type and distribution of the variables. Univariate regression analyses were performed to test the association between binomial (i.e. sex, AGM…) and continuous variables.

The predictive value of SPISE value at the baseline for the onset of abnormal glucose metabolism (IFG and/or IGT or T2D) at the follow-up was estimated by multiple logistic regression analysis adjusted for age, sex, fasting and 120 min glucose and insulin levels at the baseline. An adjusted area under receiver-operating characteristic curve (AUROC) of SPISE for AGM, with 95% confidence interval (C.I.), was also calculated controlling for the same covariates. *P* values < 0.05 were considered statistically significant with a C.I. of 95%.

## Results

### Baseline assessments

At baseline, overweight/obese children (n = 909, mean ± SD age: 10.3 ± 3.2 years; M/F 433/476, mean ± SD BMI: 27.4 ± 4.4 kg/m^2^; SDS BMI: 2.05 ± 0.4%) had mean ± SD SPISE of 6.9 ± 1.6.

In these subjects, the prevalence of alterations of glucose metabolism at baseline was almost 11% (96 out of 909 subjects). Children with IGR had significantly lower SPISE than those with normal glucose tolerance (mean ± SD SPISE: 6.3 ± 1.7*vs.*7 ± 1.6, *p* < 0.001). In our study population, triglycerides, AST, ALT, blood insulin, DI, HOMA-IR, HOMA-β%, ISI, SPISE had a skewed distribution; characteristics of the whole study population and according to the glucose tolerance profile are illustrated in Table 1.

**Table 1:** Baseline clinical characteristics of the whole study population in relation to the presence of overweight/obesity and impaired glucose metabolism

	Overweight/obese children *n* = 909	Overweight/obese NGT *n* = 813	Overweight/obese IGR *N* = 96	Normal weight children *n* = 99	*p* value
Age (years)	10.3 ± 3.2	10.2 ± 3.2	10.7 ± 3	10.5 ± 3.3	0.16° 0.59^^^
Gender (M/F)	433/476	381/432	51/45	56/43	0.23^*^°0.08^*^^
BMI (kg/m^2^)	27.4 ± 4.4	27.3 ± 4.5	28.3 ± 4.3	17.5 ± 2.8	0.04°
SDS BMI	2.05 ± 0.4	2.05 ± 0.4	2.1 ± 0.4	−0.48 ± 0.8	0.46°
Total cholesterol (mg/dL)	166.8 ± 32	166 ± 31.6	175.2 ± 31.5	164.8 ± 28.4	0.007°
HDL-C (mg/dL)	51.4 ± 12.3	51.6 ± 12.3	49.7 ± 12.5	60.2 ± 12.4	0.15°
LDL-C (mg/dL)	102.6 ± 28	103 ± 27.6	109.8 ± 29	95.2 ± 25.4	0.008°
Triglycerides (mg/dL)	62.8 ± 37.8	60.8 ± 36.4	78.5 ± 45	47.2 ± 30.9	< 0.001°
FBG (mg/dL)	90 ± 7.3	87.7 ± 5.8	99.6 ± 9.7	87.7 ± 8.6	< 0.001°
FSI (IU/mL)	15 ± 9.1	14.6 ± 9	17.9 ± 10.2	11.4 ± 5.9	0.003°
120 min BG (mg/dL)	105.3 ± 17.7	102.7 ± 14.8	126.3 ± 24.5	–	< 0.001°
120 min insulin (IU/mL)	61.5 ± 47.4	58.2 ± 44.3	89.2 ± 61.2	–	< 0.001°
SPISE	6.9 ± 1.6	7 ± 1.6	6.3 ± 1.7	13.7 ± 2.9	< 0.001°
ISI	6.9 ± 5.5	7.1 ± 5.6	4.6 ± 3.9	12.4 ± 6.8	< 0.001°
HOMA-IR	3.3 ± 2.1	3.2 ± 2	4.4 ± 2.5	2.5 ± 1.4	< 0.001°
HOMA-β%	148 ± 61.1	220.5 ± 138	186.5 ± 124.8	125.5 ± 42.3	0.002°
NGT, number (%)	813 (89.4%)	813 (100%)	0	99 (100%)	< 0.001^*^
IFG	65	0	65 (68%)	0	
IGT	27	0	27 (28%)	0	
IFG + IGT	4	0	4 (4%)	0	
DM	0	0	0	0	

Data are mean ± SD unless otherwise indicated. Differences have been compared by Student’s *T* test or *χ^2^ test, as appropriate

*BMI* body mass index, *SDS BMI* standard deviation score of body mass index, *HDL-C* high-density lipoprotein cholesterol, *LDL-C* low-density lipoprotein cholesterol, *FBG* fasting blood glucose, *FSI* fasting serum insulin, *HOMA-IR* homeostasis model assessment of insulin resistance, *HOMA-β%* homeostasis model assessment of insulin secretion, *ISI* insulin sensitivity index, *SPISE* single-point insulin sensitivity estimator, *TG/HDL* triglycerides to HDL ratio, *NGT* normal glucose tolerance, *IFG* impaired fasting glucose, *IGT* impaired glucose tolerance, *DM* diabetes mellitus

°Comparison between overweight/obese NGT vs overweight/obese IGR children

^Comparison between overweight/obese and normal-weight children

At the bivariate analysis, the SPISE index positively correlated with the ISI and DI whereas an inverse association was found between SPISE and age, blood pressure, HOMA-IR, basal and 120 min blood glucose and insulin (Table 2). The SPISE did not associate with sex at the univariate logistic analysis (*β* coefficient = 0.79, *p* = 0.19).

**Table 2:** Overweight/obese children population (*n* = 909)

	Correlation coefficient	*p* value
Age	−0.57	< 0.001
SDS BMI	−0.56	< 0.001
FBG	−0.16	< 0.001
FSI	−0.43	< 0.001
120 min BG	−0.11	< 0.001
120 min insulin	−0.36	< 0.001
Total cholesterol	−0.007	0.82
LDL-C	−0.03	0.35
ISI	0.45	< 0.001
DI	0.17	< 0.001
HOMA-IR	−0.43	< 0.001
HOMA-β	−0.35	< 0.001
SBP	−0.55	< 0.001
DBP	−0.42	< 0.001

Bivariate correlation analyses between SPISE index and clinical parameters; Pearson’s correlation coefficient

*BMI* body mass index, *SDS BMI* standard deviation score of body mass index, *HDL-C* high-density lipoprotein cholesterol, *LDL-C* low-density lipoprotein cholesterol, *FBG* fasting blood glucose, *FSI* fasting serum insulin, *HOMA-IR* homeostasis model assessment of insulin resistance, *HOMA-β%* homeostasis model assessment of insulin secretion, *ISI* insulin sensitivity index, *DI* disposition index, *SBP* systolic blood pressure, *DBP* diastolic blood pressure

The existence of a significant association between SPISE and insulin-derived indexes of insulin sensitivity (ISI), resistance (HOMA-IR) and secretion (HOMA-β%) was also detected in a cohort of 99 normal-weight metabolically healthy children (ISI: *r* = 0.55, *p* < 0.001; HOMA-IR: *r* = −0.44, *p* = 0.002; HOMA-β %: *r* = −0.4, *p* = 0.006). In this subgroup, the SPISE index negatively correlated with age (*r* = −0.43, *p* < 0.001) and FSI (*r* = −0.47, *p* < 0.001); no association with FBG (*r* = −0.08, *p* = 0.47) or sex (β = −2.2, p = 0.29) was observed.

**Fig.1:**
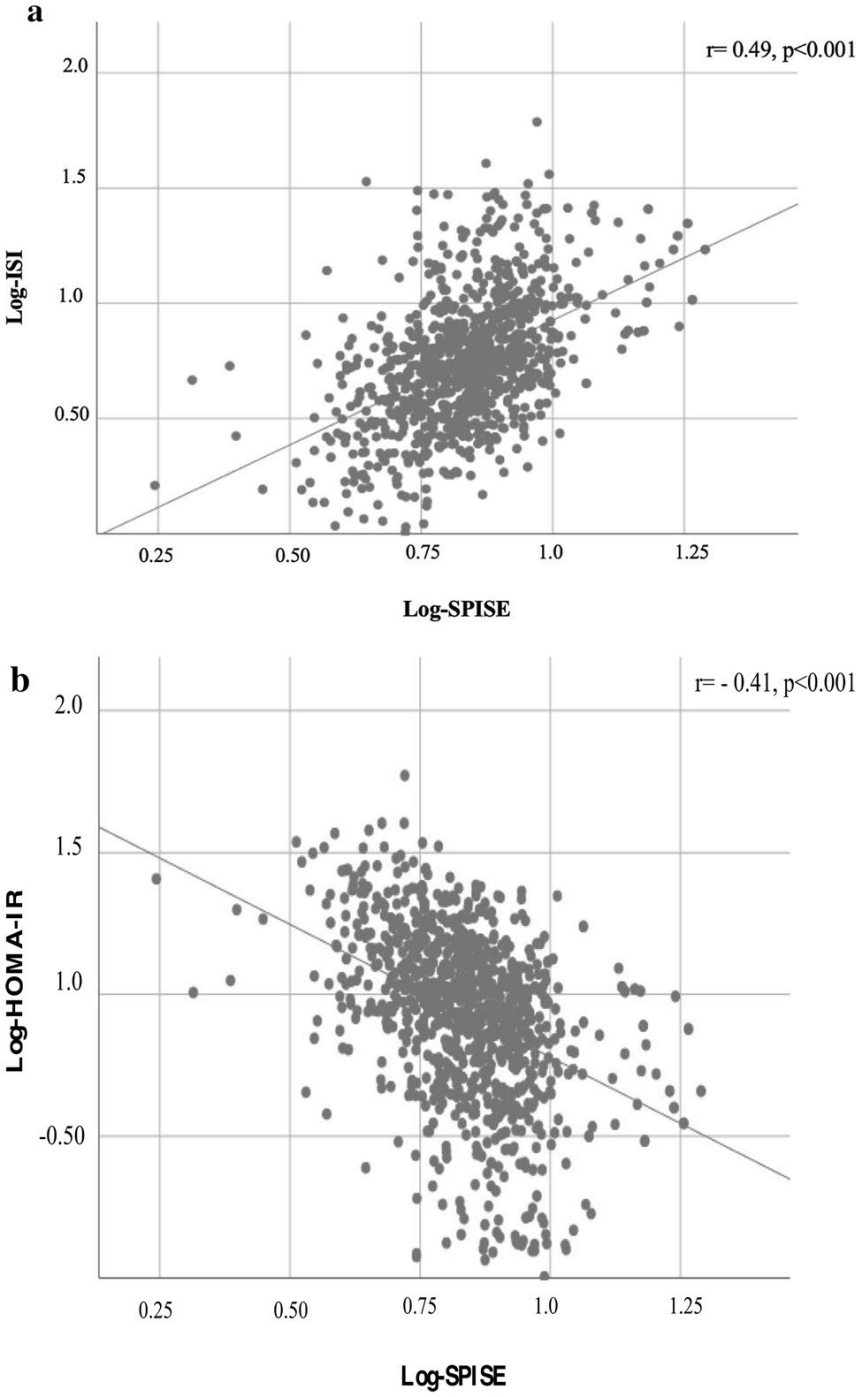
Correlation between SPISE, ISI (**a**) and HOMA-IR (**b**) in the whole study population (*n* = 1008). Pearson’s correlation coefficient calculated

Finally, the SPISE index strongly correlated with ISI (A; [*r* = 0.49, *p* < 0.001]) and HOMA-IR [B; (*r* = −0.41, *p* < 0.001)] in the whole study population of 1008 children belonging to different BMI classes (Fig. 1a, b).

### Follow-up analysis

Among the 200 overweight/obese children undergoing clinical follow-up, 31 subjects (15%) developed IGR (*n* = 21 IFG, *n* = 4 IFG + IGT, *n* = 6 IGT) at the 6.5 (3.5–10) year evaluation.

Characteristics of this study subgroup at the baseline and follow-up are illustrated in Table 3.

**Table 3:** Characteristics of the subgroup of overweight/obese children (*n* = 200) undergoing 6.5 year follow-up (baseline and end of observation)

	Baseline *n* = 200	Follow-up *n* = 200	*p* value
Age (years)	9.58 ± 3.1	16.4 ± 3.7	< 0.001
BMI (kg/m^2^)	26.48 ± 3.9	29.65 ± 5.9	< 0.001
SDS BMI	1.95 ± 0.3	1.88 ± 1.01	0.37
SBP (mmHg)	104.15 ± 13.5	114.89 ± 11.6	< 0.001
DBP (mmHg)	61.01 ± 8.5	70.98 ± 8.3	< 0.001
Total cholesterol (mg/dL)	167.5 ± 33.1	172.21 ± 34.8	0.043
HDL-C (mg/dL)	51.76 ± 12.6	50.69 ± 12.3	0.28
LDL-C (mg/dL)	103.26 ± 28.9	104.90 ± 28.9	0.36
Triglycerides (mg/dL)	58.76 ± 32.5	82.03 ± 39.7	< 0.001
FBG (mg/dL)	88.64 ± 7.3	88.64 ± 9.8	0.99
FSI (IU/mL)	13.39 ± 8.7	15.24 ± 10.96	0.08
120 min glucose (mg/dL)	105.06 ± 17.4	99.52 ± 21.9	0.003
120 min insulin (mg/dL)	55.74 ± 43.6	68.74 ± 52.2	0.007
SPISE	7.29 ± 1.7	6.01 ± 1.9	< 0.001
ISI	7.86 ± 5.9	6.37 ± 3.8	0.009
DI	74.06 ± 42.1	82.24 ± 61.99	0.13
HOMA-IR	2.96 ± 1.99	3.36 ± 2.6	0.11
HOMA-β%	197.49 ± 137.8	249.52 ± 202.9	0.005

Data are mean ± SD unless otherwise indicated. Differences compared by Student’s *T* test

*BMI* body mass index, *SDS BMI* standard deviation score of body mass index, SBP systolic blood pressure, DBP diastolic blood pressure, *HDL-C* high-density lipoprotein cholesterol, *LDL-C* low-density lipoprotein cholesterol, *FBG* fasting blood glucose, *FSI* fasting serum insulin, *SPISE* single-point insulin sensitivity estimator, *ISI* insulin sensitivity index, *DI* disposition index, *HOMA-IR* homeostasis model assessment of insulin resistance, *HOMA-β%* homeostasis model assessment of insulin secretion

Baseline SPISE index inversely correlated with age, BMI, SDS–BMI% and waist circumference at the follow-up. Having a lower SPISE index at baseline was associated with the development of higher blood pressure levels, impaired glucose and lipid profile at the follow-up evaluation (Table 4).

**Table 4:** Bivariate correlation analyses between baseline SPISE and clinical parameters at the follow-up; Pearson’s coefficient

	Correlation coefficient	*p* value
Age	−0.60	< 0.001
SBP	−0.58	< 0.001
DBP	−0.47	< 0.001
BMI	−0.45	< 0.001
SDS BMI	−0.31	0.001
Waist circumference	−0.57	< 0.001
FBG	−0.42	< 0.001
120 min BG	−0.12	0.11
FSI	−0.05	0.53
120 min insulin	−0.12	0.11
Total cholesterol	−0.16	0.02
HDL-C	0.12	0.07
LDL-C	−0.14	0.04
Triglycerides	−0.29	< 0.001
HOMA-IR	−0.12	0.11
HOMA-β	0.19	0.009
ISI	−0.01	0.90
DI	0.31	< 0.001

*SBP* systolic blood pressure, *DBP* diastolic blood pressure, *BMI* body mass index, *SDS BMI* standard deviation score of body mass index, *FBG* fasting blood glucose, *FSI* fasting serum insulin, *HDL-C* high-density lipoprotein cholesterol, *LDL-C* low-density lipoprotein cholesterol, *HOMA-IR* homeostasis model assessment of insulin resistance, *HOMA-β%* homeostasis model assessment of insulin secretion, *ISI* insulin sensitivity index, *DI* disposition index

**Fig.2:**
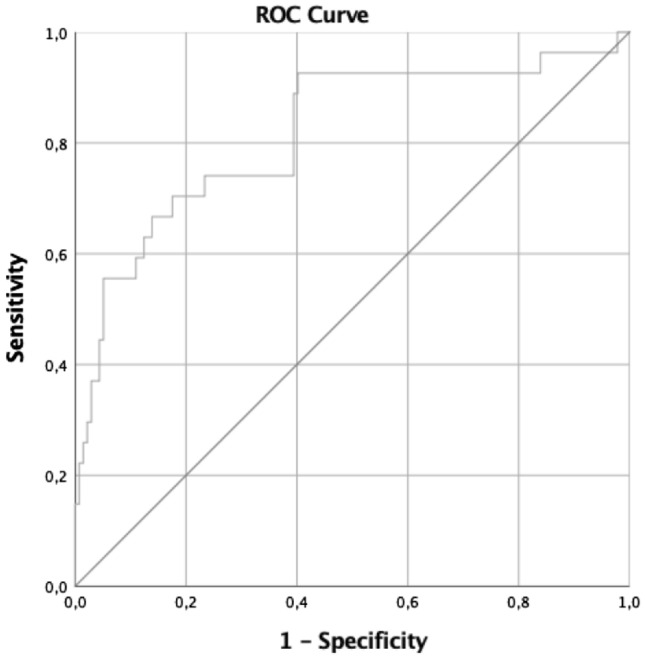
AUROC of the SPISE index for the prediction of IGR at the follow-up

Belonging to the lowest quartile of the SPISE index distribution (i.e. SPISE index below 6.08) at baseline was associated with the development of IGR later in life with OR = 3.89 (1.65–9.13); *β* = 1.36; *p* = 0.002, at the multivariate logistic regression analysis adjusted for age, sex, fasting and 120 min glucose and insulin levels at baseline. The SPISE index showed high specificity and sensitivity in predicting future IGR, with AUROC curve = 0.82(0.72–0.92), *p* < 0.001 in the adjusted AUROC model corrected for the same covariates (Fig. 2).

Notably, unlike SPISE, neither ISI nor HOMA-IR at baseline were able to predict the development of IGR in obese children later in life in multivariate logistic regression models adjusted for sex, age and BMI [ISI: OR = 0.94 (0.87–1.02), *p* = 0.12; HOMA-IR: OR 1.07 (0.85–1.34), *p* = 0.57].

## Discussion

The main finding of this study is that the SPISE index correlates with insulin-derived indicators of insulin resistance and sensitivity in children, and significantly predicts the development of glucose metabolism abnormalities later in life in this population.

Indeed, on one hand, the SPISE index displayed a strong cross-sectional correlation with dynamic OGTT-derived indicators of insulin sensitivity such as ISI, or with the widely used proxy of insulin resistance HOMA-IR. On the other, low basal SPISE was able to predict the development of impaired glucose regulation at the 6.5-year follow-up with an OR of 3.89(1.65–9.13) regardless of major metabolic confounders such as sex, age, and results from the OGTT performed at the baseline. At variance with SPISE, in our study, all the other insulin-derived indexes of insulin sensitivity/resistance calculated at baseline failed to demonstrate any correlation with future onset of IGR in children with body weight excess.

This is the first study aiming to test the reliability of the SPISE index as an indicator of insulin sensitivity in children and to assess its predictive value for the identification of glucose-insulin metabolism disorders later in life.

In the cross-sectional phase of this study, the association between the SPISE index and the OGTT-derived ISI, a validated indirect indicator of low insulin sensitivity also applied in children and adolescents [34, 35] was demonstrated in over 900 youths with body weight excess and was then confirmed in normal-weight children.

A number of studies previously investigated the relationship between the SPISE index and insulin-derived indicators of insulin homeostasis [23, 24, 36–39]; data showed that the SPISE index was comparable to Matsuda-ISI, QUICKI and HOMA-IR when used for the identification of conditions of altered insulin sensitivity in adults [23]. Moreover, lower SPISE index significantly correlated in adults or adolescents with the presence of T2D [36], metabolic syndrome [37, 39], risk of cardiovascular diseases [36], non-alcoholic fatty liver disease (NAFLD)[38], abdominal obesity, higher levels of C-reactive protein (CRP) and lower levels of adiponectin[24].

Finally, in line with our results obtained in youths, Sagesaka et al*.* demonstrated that basal SPISE index was significantly lower in adults who developed T2D 10 years later in comparison to those who did not progress to diabetes, in a longitudinal investigation on over 27,000 individuals without diabetes [40].

Our study is the first investigation testing the SPISE index in children. The rise of childhood obesity in Western countries is paralleled by the increasing prevalence of T2D and other metabolic diseases such as NAFLD and metabolic syndrome in children and pre-adolescents [41, 42], as a significant proportion of overweight/obese children is also affected by subclinical-insulin resistance [16, 18, 19].

Therefore, identifying an easy, reliable and cost-effective tool for the stratification of cardio-metabolic risk in youths with body weight excess is a primary goal to achieve for containing the burden of T2D and other metabolic complications of obesity across the new generations[43].Indeed, although the euglycaemic-hyperinsulinemic clamp represents the “gold standard” for measuring insulin sensitivity[44], this technique is invasive, expensive and difficult to perform in the clinical practice.

Thus, surrogate indexes of insulin resistance have been developed [23, 31–33]. Some of these include insulin or glucose loading and their measurement in determined time intervals, such as the Matsuda index, intravenous glucose tolerance test (IVGTT), insulin tolerance test (ITT)[45]. Other indexes consist in the measurement of insulin levels in a steady state (including HOMA-IR, QUICKI, 1/HOMA, HOMA-1%S, etc.) [45–47]. However, the value of insulin measurement and insulin-derived indicators for metabolic risk stratification in the young population is still debated, and their interpretation is not univocal due to the pulsatility in insulin release [20], its short half-life [22] and the presence of poorly standardized assays [21].

In this setting, the identification of SPISE, a lipids and BMI derived index of insulin sensitivity, as a novel predictor of impaired glucose-insulin metabolism, provides a unique tool to be used in clinical practice for phenotyping children at high risk of metabolic diseases, such as those with obesity. Moreover, since insulin measurement is not advised for screening insulin resistance in large groups or for preventive purposes [16], whereas most of the population-based health surveys include BMI and lipid profile [23], the SPISE index may also be used as a sensitive and easy method to assess insulin sensitivity at the population level.

The SPISE index has been validated in a cross-sectional investigation including a large cohort of over 1200 nondiabetic adults and 29 obese adolescents [23]. In this study, a cut-off value of SPISE below 6.61 was proposed to indicate the presence of insulin resistance, as estimated by the comparison with the clamp-derived *M* value.

Conversely, our study is the first investigation exploring the SPISE index in the prediction of impaired glucose regulation development in overweight and obese children. Thus, rather than using a previously identified SPISE cut-off obtained in a non-comparable population and study design, in our study we explored whether belonging to the lowest quartile of the SPISE index distribution, i.e. SPISE below 6.08, at baseline was associated with the development of altered glucose metabolism. Thus, a SPISE index cut-off < 6.08 may be proposed as a novel threshold for low insulin sensitivity in children which could predict the development of dysglycaemia later in life in the setting of the real world evidence.

The rationale of the SPISE index to identify insulin resistance is particularly intriguing: TG and HDL represent changes in lipids and lipoproteins that are among the earliest manifestations of insulin resistance [48–50]. Indeed, insulin resistance measured by euglycaemic clamp is associated with adverse lipid and lipoprotein changes favoring atherosclerosis even in subjects without diabetes. The addition of BMI, another easy indirect measure of adipose tissue and insulin sensitivity, further enhances the sensitivity of the SPISE index. For all these characteristics, the SPISE index, but not traditional insulin-derived indicators of insulin sensitivity/resistance such ad ISI and HOMA-IR, performed very well as a strong independent predictor of development of IGR in the large population of overweight and obese children included in this study.

In conclusion, this study demonstrates that the SPISE index is a strong indicator of insulin sensitivity in children with and without body weight excess, and that in overweight/obese individuals it predicts the development of impaired glucose regulation later in life independently from potential confounders. Finally, for its characteristics of non-invasive, low-cost and simple to estimate index, the SPISE index may represent an easy surrogate of insulin sensitivity in overweight/obese children to be used as a screening tool for metabolic risk assessment on a large scale.

## Data Availability

The authors agree to share data upon request.
